# Decreased Protein Quality Control Promotes the Cognitive Dysfunction Associated With Aging and Environmental Insults

**DOI:** 10.3389/fnins.2018.00753

**Published:** 2018-11-01

**Authors:** Hisayo Jin, Mari Komita, Tomohiko Aoe

**Affiliations:** ^1^Department of Anesthesiology, Chiba University Graduate School of Medicine, Chiba, Japan; ^2^Department of Anesthesiology, Chiba Rosai Hospital, Ichihara, Japan; ^3^Department of Medicine, Pain Center, Chiba Medical Center, Teikyo University, Ichihara, Japan

**Keywords:** neurodegeneration, aging, chaperone, endoplasmic reticulum, quality control, proteostasis, anesthetics

## Abstract

**Background:** Most neurodegenerative diseases are sporadic and develop with age. Degenerative neural tissues often contain intra- and extracellular protein aggregates, suggesting that the proteostasis network that combats protein misfolding could be dysfunctional in the setting of neurodegenerative disease. Binding immunoglobulin protein (BiP) is an endoplasmic reticulum (ER) chaperone that is crucial for protein folding and modulating the adaptive response in early secretory pathways. The interaction between BiP and unfolded proteins is mediated by the substrate-binding domain and nucleotide-binding domain with ATPase activity. The interaction facilitates protein folding and maturation. BiP has a recovery motif at the carboxyl terminus. The aim of this study is to examine cognitive function in model mice with an impaired proteostasis network by expressing a mutant form of BiP lacking the recovery motif. We also investigated if impairments of cognitive function were exacerbated by exposure to environmental insults, such as inhaled anesthetics.

**Methods:** We examined cognitive function by performing radial maze testing with mutant BiP mice and assessed the additional impact of general anesthesia in the context of proteostasis dysfunction. Testing over 8 days was performed 10 weeks, 6 months, and 1 year after birth.

**Results:** Age-related cognitive decline occurred in both forms of mice. The mutant BiP and anesthetic exposure promoted cognitive dysfunction prior to the senile period. After senescence, when mice were tested at 6 months of age and at 1 year old, there were no significant differences between the two genotypes in terms of the radial maze testing; furthermore, there was no significant difference when tested with and without anesthetic exposure.

**Conclusion:** Our data suggest that aging was the predominant factor underlying the impairment of cognitive function in this study. Impairment of the proteostasis network may promote age-related neurodegeneration, and this is exacerbated by external insults.

## Introduction

Newly synthesized polypeptides destined for secretory proteins and transmembrane proteins are inserted into the endoplasmic reticulum (ER) and monitored by the proteostasis network. Nascent peptides avoid aggregation and degradation by associating with molecular chaperones in the ER lumen, such as binding immunoglobulin protein (BiP), calreticulin, and protein disulfide isomerase (Alder et al., 2005). The interaction between secreted proteins and ER chaperones promotes their mature tertiary structure, thus facilitating their transport from the ER to the Golgi and subsequently other cellular compartments (e.g., endosomes and plasma membranes) or the extracellular space (Ellgaard and Helenius, 2003; Braakman and Hebert, 2013). Improperly folded proteins are degraded, or sequestered as aggregates inside and outside the cell, leading to stress responses such as the unfolded protein response in the ER (Ron and Walter, 2007) and the heat shock response in the cytosol (Brandvold and Morimoto, 2015; Balchin et al., 2016), which increases the capacity of the proteostasis network. Failure of this response causes cellular dysfunction and cell death in various human diseases (Walter and Ron, 2011).

Most neurodegenerative diseases occur sporadically in the middle aged to elderly, most likely because the capacity of the proteostasis network decreases with age (Brown and Naidoo, 2012; Gorbatyuk and Gorbatyuk, 2013; Labbadia and Morimoto, 2015). Protein aggregations in neuronal tissue are a hallmark of neurodegenerative conditions such as Parkinson’s disease and Alzheimer’s disease. Neuronal failure of the proteostasis network may cause protein aggregation that leads to neurodegeneration (Ogen-Shtern et al., 2016; Hetz and Saxena, 2017). BiP (GRP78) is a member of the 70-kDa heat shock protein (HSP 70) family present in the ER. Deletion of BiP is lethal to yeast cells (the *Kar2* gene in yeast) (Rose et al., 1989) and early mouse embryonic cells (Luo et al., 2006). Interactions between BiP and unfolded proteins are mediated by the substrate-binding domain and the nucleotide-binding domain of ATPase activity. The interaction facilitates protein folding and maturation. For mice or humans, mutations in *Sil1* genes, which encode an adenine nucleotide exchange factor for BiP, cause various degrees of neurodegeneration (Senderek et al., 2005; Zhao et al., 2005). BiP also has a KDEL (Lys-Asp-Glu-Leu) sequence at the carboxyl terminus (Munro and Pelham, 1987). When BiP is secreted from the ER with associated unfolded proteins, the KDEL receptor in the post-ER compartment recognizes BiP’s KDEL sequence and transfers the protein and its cargo to the ER via coat protein complex I (COPI) vesicle transport (Yamamoto et al., 2001).

Yeast studies revealed that recovery of BiP by this receptor was not essential in a single cell (Beh and Rose, 1995). Mutant BiP with deleted KDEL is functional as an ER chaperone, since mouse embryonic fibroblasts derived from the homozygous mutant BiP mice are also viable. Upon secretion from the ER carrying unfolded proteins, mutant BiP is not recognized by the KDEL receptor in the Golgi and it is, therefore, not recovered to the ER, leading to protein mis-sorting (Yamamoto et al., 2001). Such unfolded proteins, when secreted in association with mutant BiP, may not undergo sufficient maturation in the early secretory pathway. In fact, experiments in knock-in mice expressing mutant BiP with a deleted retrieval KDEL motif revealed that some essential proteins, like surfactants and reelin, require KDEL receptor-mediated retrieval of BiP for folding and maturation (Mimura et al., 2007; Mimura et al., 2008). Notably, homozygous mutant BiP mice die soon after birth due to respiratory failure. Furthermore, homozygous mutant BiP mice show dysregulated neuronal development. In wild-type mice, neurosecretory Cajal–Retzius cells are located in the superficial layer of the cortex. However, those in mutant BiP mice are scattered around the upper layer of the neocortical primordium. Cajal–Retzius cells in the BiP mutant do not secrete reelin. Although mRNA levels are normal, the expression of reelin protein is reduced.

The lifespan of heterozygous mutant BiP knock-in mice is not significantly different from wild-type mice, but various pathological conditions can manifest. We have previously observed kidney dysfunction and motor disorder as mutant mice age (Kimura et al., 2008; Jin et al., 2014). Some of the mutant BiP mice also displayed paralysis and tremors after 12 months. Some large motoneurons express C/EBP homologous protein (CHOP), a cell death-related transcriptional factor during ER stress. In accordance with these findings, TUNEL staining identified several apoptotic cells and enhanced gliosis in the mutant spinal cord (Jin et al., 2014).

Memory impairment is an early symptom of several neurodegenerative conditions including Alzheimer’s disease. However, cognitive dysfunction may be difficult to recognize on its own, not only in humans but also in mice. In this study, we examined cognitive function by performing radial maze testing with mutant BiP mice and assessed the additional impact of general anesthesia in the context of proteostasis dysfunction. Long-term observations revealed that cognitive decline due to aging is more rapid in mutant BiP mice compared to wild-type mice. In addition, exposure to inhaled anesthetics is common in modern society, and this exacerbated cognitive impairment in mutant mice. Collectively, our results suggest that reduced capacity of the proteostasis network, where BiP plays a significant role, may contribute to age-associated neurodegeneration.

## Materials and Methods

### Animals

This study was carried out in accordance with the recommendations of the guidelines for animal experiments of Chiba University. The protocol was approved by the Institutional Animal Care Committee of Chiba University, Chiba, Japan. Generation of the knock-in mouse expressing a mutant BiP lacking the carboxyl-terminal KDEL sequence was described previously (Mimura et al., 2007). The missing KDEL sequence was replaced with a hemagglutinin (HA) tag. The heterozygous mutant BiP male mice (Bm/+) were mated with wild-type (C57BL/6,+/+) female mice, and offspring (wild-type and heterozygous) were bred for use in experiments. Subsets of the groups underwent eight-arm radial maze testing at 10 weeks, 6 months, and 12 months after birth (+/+, *n* = 6; Bm/+; *n* = 8).

### Inhalational Anesthetic Exposure

Heterozygous mutant BiP male mice were mated with wild-type (C57BL/6) female mice, and the pregnant mice were exposed to 3% sevoflurane for 3 h 2 days prior to parturition as previously described (Komita et al., 2013). The heterozygotes and wild-type newborn mice with anesthetic exposure underwent eight-arm radial maze testing at 10 weeks, 6 months, and 12 months after birth (S+/+, *n* = 12; SBm/+; *n* = 16).

### Eight-Arm Radial Maze Test

Mice were trained for 3 days before the experiment and learned the location of the food reward. Water was freely available. Fasting began at 18:00, and the experiment started at 10:00 the following day. A mouse was placed in the center of the eight-arm radial maze (SRM-M, Bio-research Center, Nagoya, Japan). The mouse freely explored and ate the food rewards placed at the arm tips. The trial time was limited to 10 min. Animal movements were recorded and evaluated blindly later. The total time for the mouse to eat all the food in the eight arms was the completion time (seconds). Attempts to enter arms without bait were counted as errors, while attempts to enter arms with food were counted as successes. The ratio of successful attempts to overall attempts was the correct answer rate (0.0–1.0). Testing was carried out daily for 8 days (Supplementary Datasets S1, S2).

### Immunohistochemistry

Mice were deeply anesthetized with pentobarbital (Dainippon Sumitomo Pharma, Osaka, Japan) and washed by transcardiac perfusion with phosphate-buffered saline (PBS). Brains were immersed and fixed for 24 h in 4% paraformaldehyde at 4°C. After fixation, brains were dehydrated in increasing concentrations of ethanol and embedded in paraffin wax. Brain sections (8 μm) were incubated with 10% normal goat serum in PBS for 30 min to block non-specific antibody binding and then incubated with a rabbit antiserum against ubiquitin (Santa Cruz Biotechnology, Santa Cruz, CA, United States) in PBS for 12 h at 4°C. Sections were rinsed with PBS, incubated with secondary antibody in PBS for 2 h at room temperature, and then visualized using a Vectastain Elite ABC kit (Vector Laboratories, Burlingame, CA, United States) with diaminobenzidine (Sigma Chemical, St. Louis, MO, United States). The signal was observed under a microscope using an N-Achroplan 406 NA 0.65 objective lens (Axio Imager A1, Carl Zeiss, Oberkochen, Germany). Luminance and contrast were optimized by AxioVision Rel. 4.7 software (Carl Zeiss), and images were taken with a digital camera (AxioCam MRc, Carl Zeiss).

### Western Blotting

Mice were deeply anesthetized with pentobarbital and washed by transcardiac perfusion with PBS. Spinal cords and brains were homogenized by sonication (UR-20P, TOMY, Tokyo, Japan) in buffer containing 0.4% (w/v) Nonidet P-40, 0.2% N-lauroylsarcosine, 30 mM Tris/HCl pH 8.0, 1 mM EDTA, 10 μg/ml aprotinin, 10 μg/ml leupeptin, 1 mM sodium orthovanadate, and 30 μg/ml N-acetyl-L-leucinal-L-lecinal-L-norleucinal. The lysates were centrifuged, and the supernatants were resuspended in SDS sample buffer and then separated by SDS-polyacrylamide gel electrophoresis under reducing conditions. The proteins were transferred from the gels to polyvinylidene fluoride membranes (Immobilon-P, Millipore Corp., Billerica, MA, United States), and Western blotting was performed as previously described (Jin et al., 2014). The following antibodies were used: a rabbit antiserum against ubiquitin (Santa Cruz Biotechnology), a mouse mAb SPA-827 against BiP (KDEL sequence) (Stressgen, Victoria, BC, Canada), and a mouse mAb against γ-tubulin (Sigma Chemical). Imaging was performed with an LAS-1000 Image Analyzer and Image Gage software (Fuji Photo Film Co., Ltd., Tokyo, Japan) (Supplementary Figure S1).

### Statistical Analysis

The radial maze test results are shown as means + standard error of the mean (SEM). Repeated measures analyses of variance (ANOVAs), followed by Bonferroni multiple comparison tests, were performed to compare values between groups (GraphPad Prism 4.0, GraphPad Software, San Diego, CA, United States). Statistical significance was accepted at *P* < 0.05.

## Results

**FIGURE 1:**
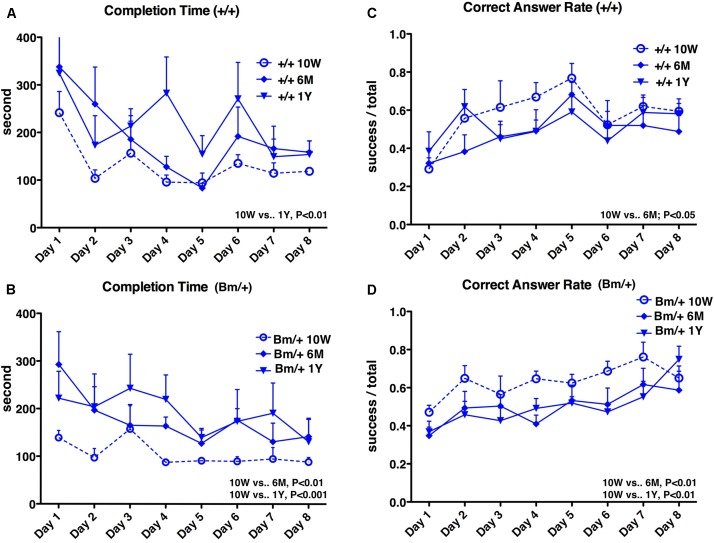
Spatial working memory is impaired with age. Eight-arm radial maze test was performed on wild-type mice (**A**,**C**,+/+; *n* = 6) and mutant-BiP mice (**B**,**D**, Bm/+; *n* = 8) at 10 weeks (10W), 6 months (6M), and 1 year (1Y) after birth. Tests were carried out for 8 days at each time point. Completion times **(A,B)** are shown as the mean time (seconds) of each group + SEM. Differences were determined by one-way repeated measures ANOVA followed by Bonferroni’s multiple comparison testing. **(A)**+/+; ANOVA, *P* = 0.0050; 10 weeks vs. 1 year, *P* < 0.01. **(B)** Bm/+; ANOVA, *P* = 0.0001; 10 weeks vs. 6 months, *P* < 0.01; 10 weeks vs. 1 year, *P* < 0.001. Correct answer rates **(C,D)** are shown as the mean of each group + SEM. The ratio of successful attempts to overall attempts was the correct answer rate (0.0–1.0). Differences were determined by one-way repeated measures ANOVA followed by Bonferroni’s multiple comparison testing. **(C)**+/+; ANOVA, *P* = 0.0412. 10 weeks vs. 6 months; *P* < 0.05. **(D)** Bm/+; ANOVA, *P* = 0.0006; 10 weeks vs. 6 months, *P* < 0.01; 10 weeks vs. 1 year, *P* < 0.01.

Radial maze testing was used to evaluate how cognitive functions were affected by aging in the setting of proteostasis dysfunction. The maze has eight arms radiating out from a central platform and is widely used to test murine spatial working memory (Olton and Papas, 1979). Testing over 8 days was performed 10 weeks, 6 months, and 1 year after birth. Learning effects (shorter completion times and higher correct answer rates) were observed in general within each 8-day period. In wild-type mice, the completion time was shortest at 10 weeks after birth and significantly longer at 1 year (*P* < 0.01). In heterozygous mutant BiP mice, the completion time was also shortest at 10 weeks, but was already significantly longer at 6 months (*P* < 0.01); this was more obvious at 1 year (*P* < 0.001) (Figures 1A,B). In wild-type mice, the correct answer rate was highest at 10 weeks, but there was no significant difference between 10 weeks and 1 year. In heterozygous mutant BiP mice, the correct answer rate was highest at 10 weeks and significantly decreased at both subsequent time points (both *P* < 0.01) (Figures 1C,D). Overall, mutant BiP mice tended to show an earlier decline in cognitive function compared to wild-type littermates.

**FIGURE 2:**
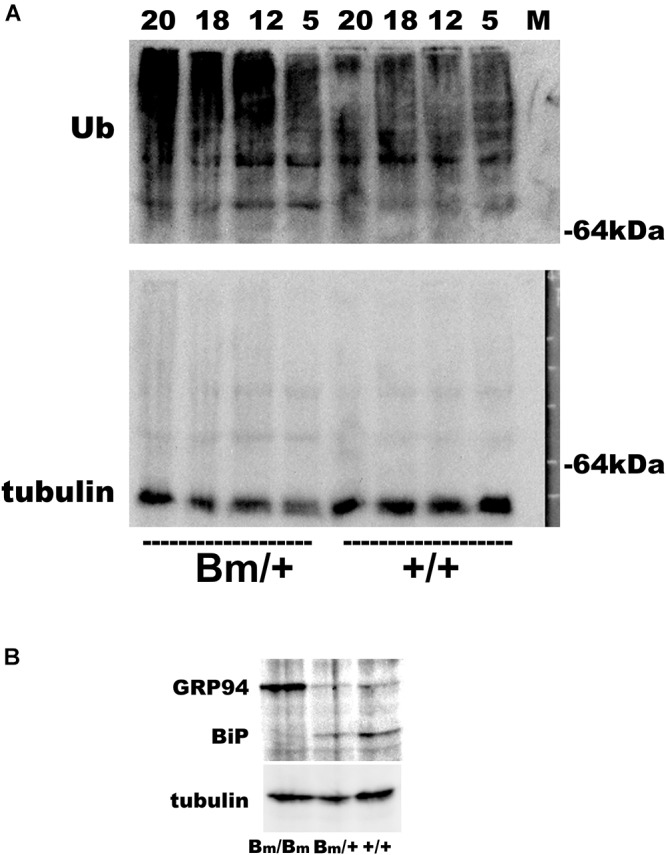
Ubiquitinated proteins are more obvious in the spinal cord of mutant BiP mice. **(A)** Spinal cords from wild-type mice (+/+, 5, 12, 18, and 20 months old) and littermate heterozygous mutant BiP mice (Bm/+, 5, 12, 18, and 20 months old) were subjected to western blot analysis with an anti-ubiquitin or an anti-tubulin antibody followed by the secondary antibody. **(B)** Brains from wild-type neonate (+/+), and heterozygous (Bm/+) and homozygous (Bm/Bm) mutant BiP neonates were subjected to western blot analysis with an anti-KDEL or an anti-tubulin antibody. The anti-KDEL antibody recognizes the KDEL sequence of BiP as well as GRP94.

**FIGURE 3:**
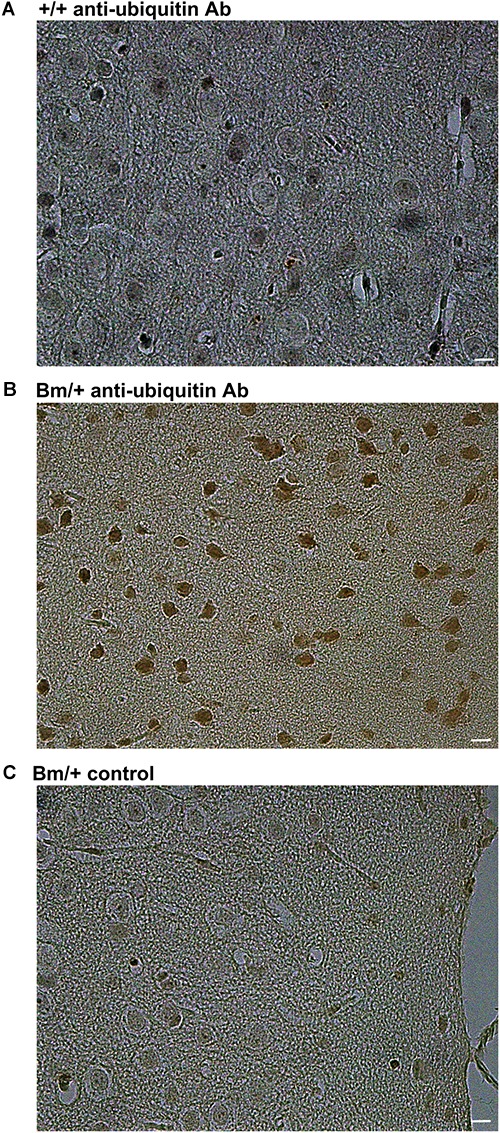
Ubiquitinated proteins are more obvious in the cerebral cortex of mutant BiP mice. Cortical tissue from **(A)** a wild-type mouse (+/+, 24 months old) and **(B)** a littermate mutant BiP mouse (Bm/+, 24 months old), which was stained with an anti-ubiquitin antibody followed by a secondary antibody. **(C)** As a negative control, cortex from a mutant BiP mouse (Bm/+, 24 months old) was incubated with saline followed by the secondary antibody. Scale bars, 10 μm.

We previously reported that heterozygous mutant BiP mice develop motor disabilities with age. Ubiquitinated protein aggregates accumulate in spinal cord motoneurons in aged mutant BiP mice (Jin et al., 2014). Western blotting of spinal cord samples from the mutant BiP mice revealed ubiquitinated proteins in older mice (Figure 2). Immunohistochemical staining of the cerebral cortex also revealed ubiquitinated protein accumulation in the mutant, suggesting the failure of proteostasis (Figure 3).

**FIGURE 4:**
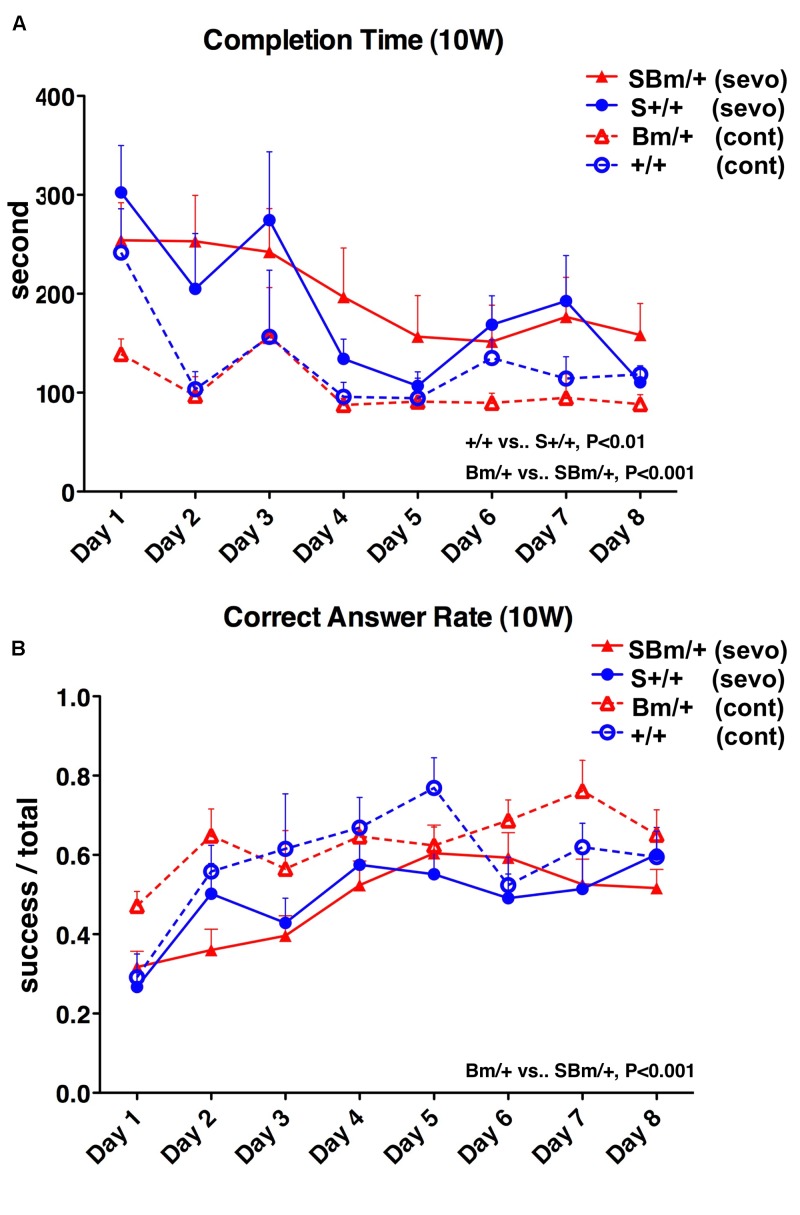
Exposure to inhalational anesthetics had more negative effects on spatial working memory in young mutant BiP mice at 10 weeks. Eight-arm radial maze testing was performed in wild-type mice (+/+; blue lines) and mutant-BiP mice (Bm; red lines) at 10 weeks after fetal anesthetic sevoflurane (S) exposure (S+/+, *n* = 12, closed circle; SBm, *n* = 16, closed triangle) and without exposure (+/+, *n* = 6, open circle; Bm, *n* = 8; open triangle). Tests were performed over 8 days. Significance was determined by one-way repeated measures ANOVA followed by Bonferroni’s multiple comparison testing. **(A)** Completion times are the average of each group + SEM. ANOVA, *P* < 0.0001; +/+10 weeks vs. +/+ S 10 weeks, *P* < 0.01; Bm/ + 10 weeks vs. SBm/ + 10 weeks, *P* < 0.001. **(B)** Correct answer rates are shown as the average value of each group + SEM. The ratio of successful attempts to overall attempts was the correct answer rate (0.0–1.0). ANOVA, *P* = 0.0002; +/+10 weeks vs. +/+S10 weeks, not significant; Bm/ + 10 weeks vs. SBm/ + 10 weeks, *P* < 0.001.

We, therefore, investigated whether an external insult would exacerbate cognitive dysfunction when the proteostasis network was impaired. General anesthesia is a common medical treatment in modern society, but could also represent an invasion. Pregnant wild-type mice mated with male heterozygous BiP mutants were exposed to the inhalation anesthetic sevoflurane. Two days prior to delivery (E17), they were exposed to 3% sevoflurane (1.2 minimal alveolar concentration in mice) for 3 h (Puig et al., 2002). Pregnant mice tolerated general anesthesia well in terms of blood pressure, tail blood flow, and percutaneous oxygen saturation (Komita et al., 2013). In our previous study, exposure to 3% sevoflurane for 3 h on E17 caused neuronal death in the homozygous mutant BiP neonatal brain in the acute phase, while no significant neuronal death was observed in heterozygotes or wild-type littermates (Komita et al., 2013). The heterozygotes and wild-type newborn mice with anesthetic exposure appeared unaffected after birth and continued breeding with their mother. Radial maze testing was performed at 10 weeks, 6 months, and 1 year. Radial maze testing at 10 weeks revealed that heterozygous mutant BiP mice with fetal anesthetic exposure had a significantly longer completion time (*P* < 0.001) and had a lower correct answer rate (*P* < 0.001) compared to unexposed mice. Exposed wild-type mice showed a significant increase in the completion time at 10 weeks (*P* < 0.01), but there was no significant difference in the correct answer rate compared to unexposed mice (Figure 4). After aging, when mice were 6 months or 1 year old, there were no significant differences in the radial maze testing between the mice of either genotype with and without anesthetic exposure (Supplementary Figures S2, S3).

**FIGURE 5:**
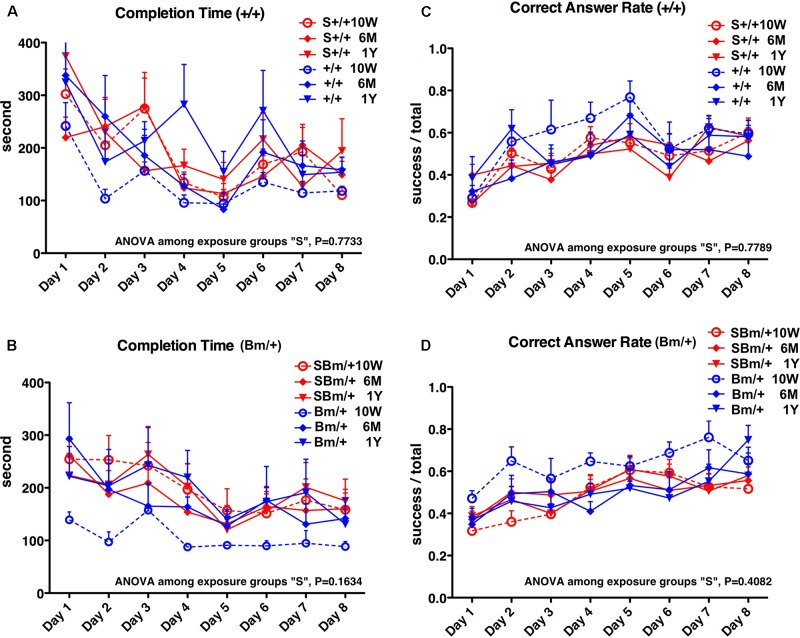
Exposure to inhalational anesthetics impaired spatial working memory in young mice. Eight-arm radial maze testing was performed in wild-type mice **(A,C)** and mutant-BiP mice **(B,D)** at 10 weeks, 6 months, and 1 year after birth. Tests were performed over 8 days at each time point. Completion times are shown as the average time (seconds) of each group + SEM. Differences among exposure groups were determined by one-way repeated measures ANOVA. **(A)** Wild-type mice with fetal anesthetic exposure (S+/+, *n* = 12; red lines) and without exposure (+/+, *n* = 6, blue lines same as Figure 1). ANOVA among exposure groups, *P* = 0.7733. **(B)** Mutant-BiP mice with fetal anesthetic exposure (SBm/+, *n* = 16; red lines) and without exposure (Bm/+, *n* = 8, blue lines same as Figure 1). ANOVA among exposure groups, *P* = 0.1634. Completion times are the average time (seconds) of each group + SEM. Differences among exposure groups were determined by one-way repeated measures ANOVA. **(C)** Wild-type mice with fetal anesthetic exposure (S+/+; red lines) and without exposure (+/+; blue lines same as Figure 1). ANOVA among exposure groups, *P* = 0.7789. **(D)** Mutant-BiP mice with fetal anesthetic exposure (SBm/+; red lines) and without exposure (Bm/+, blue lines same as Figure 1). ANOVA among exposure groups, *P* = 0.4082. The ratio of successful attempts to overall attempts was the correct answer rate (0.0–1.0).

In wild-type mice, maze completion time was prolonged at 10 weeks compared to mice without exposure, and there were no significant differences among the three time points after exposure (*P* = 0.7733) (Figure 5A, red lines). In heterozygotes, the completion time was also longer at 10 weeks compared to mice without exposure, and there were no significant differences among the three time points after exposure (*P* = 0.1634) (Figure 5B, red lines). In wild-type mice, there were no significant differences in the correct answer rate among the three time points after exposure (*P* = 0.7789) (Figure 5C, red lines). The correct answer rate in heterozygotes was reduced at 10 weeks compared to mice without exposure, and there were no significant differences among the three time points after exposure (*P* = 0.4082) (Figure 5D, red lines).

Collectively, these observations suggest that aging is the predominant factor that impairs cognitive function. This study also showed that insults, such as general anesthesia, may exacerbate cognitive decline and that individuals with impaired proteostasis may experience more significant effects.

## Discussion

We considered three factors: age, genotype, and anesthetic exposure. After senescence, when mice were 6 months and 1 year old, there were no significant differences in the radial maze testing between the two genotypes of mice tested, and between mice with and without anesthetic exposure (Supplementary Figures S2, S3). These results suggest that aging was the predominant factor responsible for the impairment of cognitive function in this study. Figures 1, 4 show that mutant BiP and anesthetic exposure promoted cognitive dysfunction, even before old age.

Protein quality control in the early secretory pathway is critical for maintaining cell homeostasis and ensuring appropriate adaptive responses to external invasion. BiP is an essential component in this process (Hendershot, 2004; Casas, 2017), as it chaperones the folding of nascent peptides in the ER. Under resting conditions, BiP associates with inositol requiring kinase-1 (IRE1), PKR-like ER related kinase (PERK), and activated transcription factor 6 (ATF6). When misfolded proteins accumulate under ER stress, BiP dissociates from ER transmembrane proteins and interacts with misfolded proteins, preventing their aggregation and facilitating their folding or degradation. This also activates ATF6, IRE1, and PERK, leading to initiation of the unfolded protein response (Bertolotti et al., 2000; Ron and Walter, 2007). Production of ER chaperones, and various proteins involved in the secretory pathway, is increased, and the capacity for quality control is expanded (Bertolotti et al., 2000; Ellgaard and Helenius, 2003; Walter and Ron, 2011). In addition, some BiP bound to misfolded proteins is secreted from the ER (Hammond and Helenius, 1994) and recognized by the KDEL receptor in the Golgi (Yamamoto et al., 2001). This activates various molecules such as hetero-trimetric G proteins (Giannotta et al., 2012; Solis et al., 2017), Src kinase (Bard et al., 2003), protein kinase A (Cabrera et al., 2003), ADP-ribosylation factor GTPase-activating protein 1 (Aoe et al., 1997; Aoe et al., 1998), and mitogen-activated protein kinases (Yamamoto et al., 2003), followed by enhanced bidirectional transport between the ER and Golgi, and transport from the Golgi to the cell surface (Cancino et al., 2014). Activation of the KDEL receptor may also play a central role in the unfolded protein response as well as the adaptive response of the intracellular transport system for external invasions (Jin et al., 2017).

Most neurodegenerative diseases occur sporadically in old age (Ogen-Shtern et al., 2016). The ability of the proteostasis network to handle protein aggregations decreases with aging (Brown and Naidoo, 2012; Gorbatyuk and Gorbatyuk, 2013; Labbadia and Morimoto, 2015). The expression and function of ER chaperones participating in proteostasis networks have been shown to decline with age (Brown and Naidoo, 2012). In fact, older heterozygous mutant BiP mice develop renal tubular-interstitial lesions and motor disabilities (Kimura et al., 2008; Jin et al., 2014). Here, we observed ubiquitinated protein accumulation in the brain and the spinal cord of aged mutant BiP mice. When the KDEL sequence was deleted, mutant BiP bound to misfolded proteins was not recovered, resulting in impaired protein folding in the early secretory pathway. Furthermore, loss of this sequence may prevent the KDEL receptor from activating autophagy (Wang et al., 2011). We found that the lack of the KDEL sequence might affect autophagy, resulting in cytosolic protein aggregations related to the late onset of motoneuron degeneration in the previous study (Jin et al., 2014).

Radial maze testing revealed decreased cognitive function in 6-month-old heterozygotes. Mouse models for neurodegeneration, such as Tau transgenic mice (Zhang et al., 2004) and amyloid precursor protein transgenic mice (Lewis et al., 2001), show definitive neurodegenerative phenotypes because they express several copies of the exogenous transgene. The mutant BiP mouse is a heterozygote with the KDEL coding sequence deleted from BiP. As with patients with sporadic neurodegenerative disease, it may be difficult to observe abnormalities in younger mutant BiP mice until they are older. Interestingly, external insults, such as proteinuria, exacerbated renal tubular injury in the aged BiP mice (Kimura et al., 2008). We, therefore, examined if exposure to inhaled anesthetics would have a significant effect on cognitive function in this model.

General anesthesia is commonly administered in modern society. Exposing very young or old individuals to inhalational anesthetics might cause neuronal degeneration followed by cognitive dysfunction, as shown in rodents and primates (Jevtovic-Todorovic et al., 2003; Raper et al., 2015; Jevtovic-Todorovic, 2016). Early clinical results suggest that children exposed to anesthetics might have impaired neuronal development and later cognitive impairment (Backman and Kopf, 1986; Wilder et al., 2009; Block et al., 2012; Ing et al., 2012; Sprung et al., 2012; Backeljauw et al., 2015). While recent comprehensive clinical studies with appropriate controls did not find a statistically significant effect of a single short-term anesthetic exposure on cognitive function (Davidson et al., 2016; Sun et al., 2016), further clinical studies that include repeated exposures, a prolonged exposure, and fragile subgroups, are necessary to clarify the effects on the brain (Vutskits and Davidson, 2017).

Exposure to inhaled anesthetics induces calcium release from the ER into the cytosol (Jevtovic-Todorovic, 2016). Aberrant calcium mobilization may alter the ER protein-folding environment to cause ER stress, leading to neurotoxicity (Wei, 2011; Chen et al., 2013; Liu et al., 2017; Zhu et al., 2017). In our previous study, we observed that exposing mice to an inhalational anesthetic enhanced expression of ER chaperones and CHOP, a transcriptional factor that induces cell death under ER stress (Komita et al., 2013). More cell death was observed in the brain of homozygous mutant BiP neonates compared to wild type mice and heterozygotes in the acute phase after sevoflurane exposure, but the long-term cognitive effects were not investigated. This study shows that sevoflurane exposure early in development significantly impaired cognitive function in both wild-type mice and BiP heterozygotes. The cognitive functional advantages of young mice were lost at 10 weeks. Anesthetic exposure had more profound effects on mice expressing BiP lacking the KDEL sequence. General anesthetics may have more dramatic detrimental effects in the developing and aging brain (Jiang and Jiang, 2015).

There are some limitations to this study which need to be considered. It is difficult to perform experiments using aged mice, especially genetic mutant mice. Although we observed littermates, the sample sizes in each group were not necessarily the same. Since this study used our original mutant mice with long-term observation, we could not fully anticipate outcomes. We had expected at first that fetal exposure to anesthetics might affect mortality, but early death was not observed. While aged mice are less homogenous, we may expose aged mice to anesthetics in future studies.

Most neurodegenerative diseases are sporadic and affect elderly populations. This study shows that aging is an important factor in cognitive decline. Our findings also suggest that patients with certain conditions characterized by defective proteostasis may be particularly vulnerable to insults such as inhaled anesthetics, ischemia, hypoxia, and toxic substances.

## Data Availability

The datasets for this study can be found in Supplementary Datasets S1–S2.

## Author Contributions

TA designed the research. HJ, MK, and TA performed the research. HJ, MK, and TA analyzed the data. TA wrote the paper.

## Conflict of Interest Statement

The authors declare that the research was conducted in the absence of any commercial or financial relationships that could be construed as a potential conflict of interest.

## Supplementary Material

FIGURE S1 **(A)** Raw image for Figure 2A, Ub. **(B)** Raw image for Figure 2A, tubulin. **(A)** and **(B)** are the same membrane, which was sequentially probed with an anti-ubiquitin and an anti-tubulin antibody. **(C)** Raw images for Figure 2B, BiP. The anti-KDEL antibody recognizes the KDEL sequence of BiP as well as GRP94. **(D)** Raw images for Figure 2B, tubulin. One membrane is divided into two according to the molecular weights of BiP and tubulin.

FIGURE S2 Eight-arm radial maze testing was performed in wild-type mice (+/+; blue lines) and mutant-BiP mice (Bm; red lines) at 6 months after fetal anesthetic exposure (S+/+, *n* = 12, closed circle; SBm, *n* = 16, closed triangle) and without exposure (+/+, *n* = 6, open circle; Bm, *n* = 8; open triangle). Tests were performed on 8 days. Significance was determined by one-way repeated measures ANOVA followed by Bonferroni’s multiple comparison testing. **(A)** Completion times are shown as the average value of each group + SEM. ANOVA, *P* = 0.8310 **(B)** Correct answer rates are shown as the average value of each group + SEM. The ratio of successful attempts to overall attempts was the correct answer rate (0.0–1.0). ANOVA, *P* = 0.8516.

FIGURE S3 Eight-arm radial maze testing was performed in wild-type mice (+/+; blue lines) and mutant-BiP mice (Bm; red lines) at 1 year after fetal anesthetic exposure (S+/+, *n* = 9, closed circle; SBm, *n* = 14, closed triangle) and without exposure (+/+, *n* = 6, open circle; Bm, *n* = 8; open triangle). Tests were performed on 8 days. Significance was determined by one-way repeated measures ANOVA followed by Bonferroni’s multiple comparison testing. **(A)** Completion times are shown as the average value of each group + SEM. ANOVA, *P* = 0,7069 **(B)** Correct answer rates are shown as the average value of each group + SEM. The ratio of successful attempts to overall attempts was the correct answer rate (0.0–1.0). ANOVA, *P* = 0.5103.

DATASET S1 Eight-arm radial maze test was performed on wild-type mice (+/+; *n* = 6) and mutant-BiP mice (Bm/+; *n* = 8) at 10 weeks (10W), 6 months (6M), and 1 year (1Y) after birth. The total time for the mouse to eat all the food in the eight arms was the completion time (seconds). Attempts to enter arms without bait were counted as errors, while attempts to enter arms with food were counted as successes. The ratio of successful attempts to overall attempts was the correct answer rate (0.0–1.0). Testing was carried out daily for 8 days.

DATASET S2 Eight-arm radial maze testing was performed in wild-type mice (S+/+, *n* = 12) and mutant-BiP mice (SBm/+, *n* = 16) at 10 weeks, 6 months, and 1 year after fetal anesthetic exposure. The total time for the mouse to eat all the food in the eight arms was the completion time (seconds). Attempts to enter arms without bait were counted as errors, while attempts to enter arms with food were counted as successes. The ratio of successful attempts to overall attempts was the correct answer rate (0.0–1.0). Testing was carried out daily for 8 days.
