# Supplementary figures and images for: Decreased Protein Quality Control Promotes the Cognitive Dysfunction Associated With Aging and Environmental Insults
Source: Front Neurosci. 2018 Nov 1;12:753. doi: 10.3389/fnins.2018.00753 (PMC6221900; doi:10.3389/fnins.2018.00753)

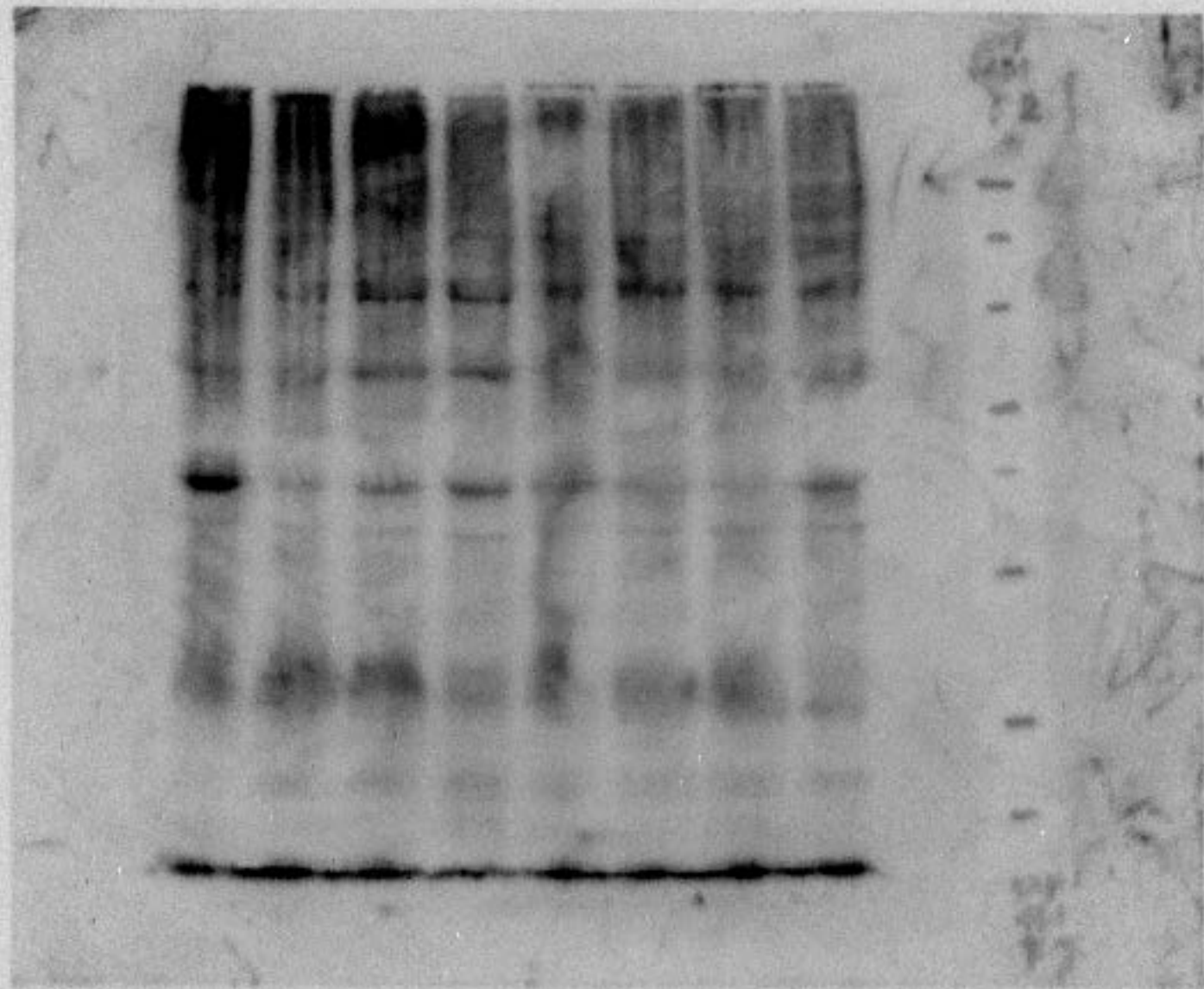

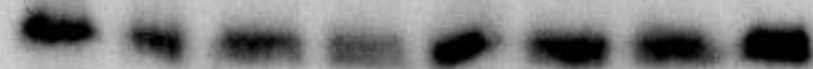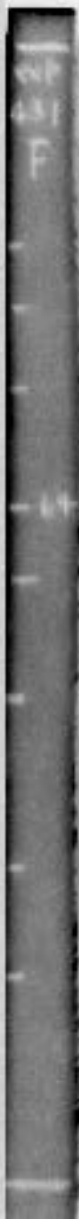

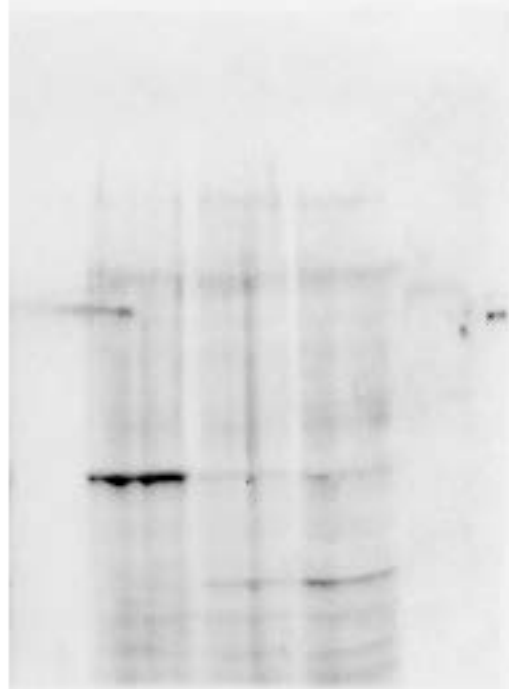

—

—

Supplement: FIGURE S1 — (A) Raw image for Figure 2A, Ub. (B) Raw image for Figure 2A, tubulin. (A) and (B) are the same membrane, which was sequentially probed with an anti-ubiquitin and an anti-tubulin antibody. (C) Raw images for Figure 2B, BiP. The anti-KDEL antibody recognizes the KDEL sequence of BiP as well as GRP94. (D) Raw images for Figure 2B, tubulin. One membrane is divided into two according to the molecular weights of BiP and tubulin. [file Image_1.pdf]

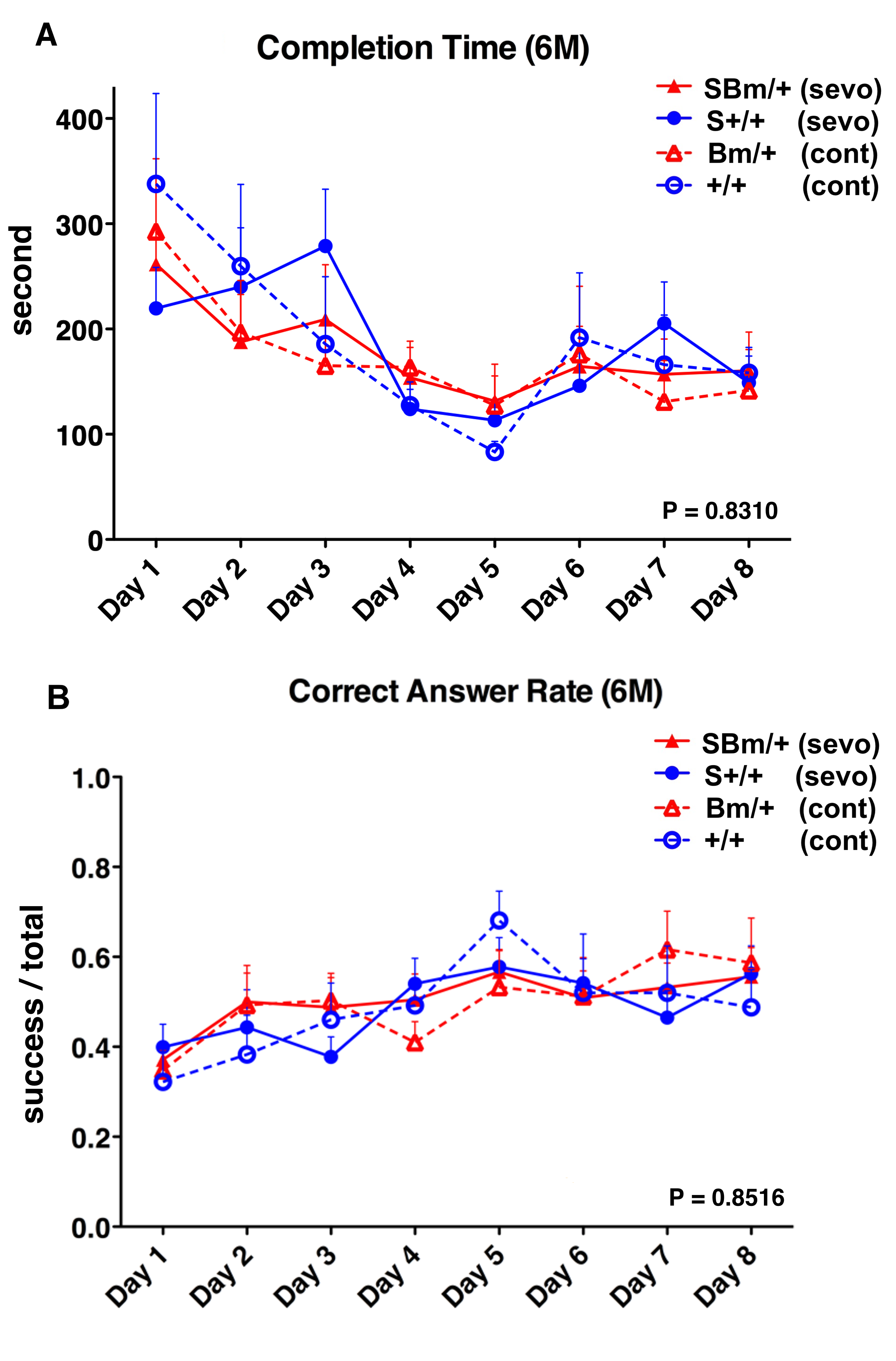

Supplement: FIGURE S2 — Eight-arm radial maze testing was performed in wild-type mice (+/+; blue lines) and mutant-BiP mice (Bm; red lines) at 6 months after fetal anesthetic exposure (S+/+, n = 12, closed circle; SBm, n = 16, closed triangle) and without exposure (+/+, n = 6, open circle; Bm, n = 8; open triangle). Tests were performed on 8 days. Significance was determined by one-way repeated measures ANOVA followed by Bonferroni’s multiple comparison testing. (A) Completion times are shown as the average value of each group + SEM. ANOVA, P = 0.8310 (B) Correct answer rates are shown as the average value of each group + SEM. The ratio of successful attempts to overall attempts was the correct answer rate (0.0–1.0). ANOVA, P = 0.8516. [file Image_2.tiff]

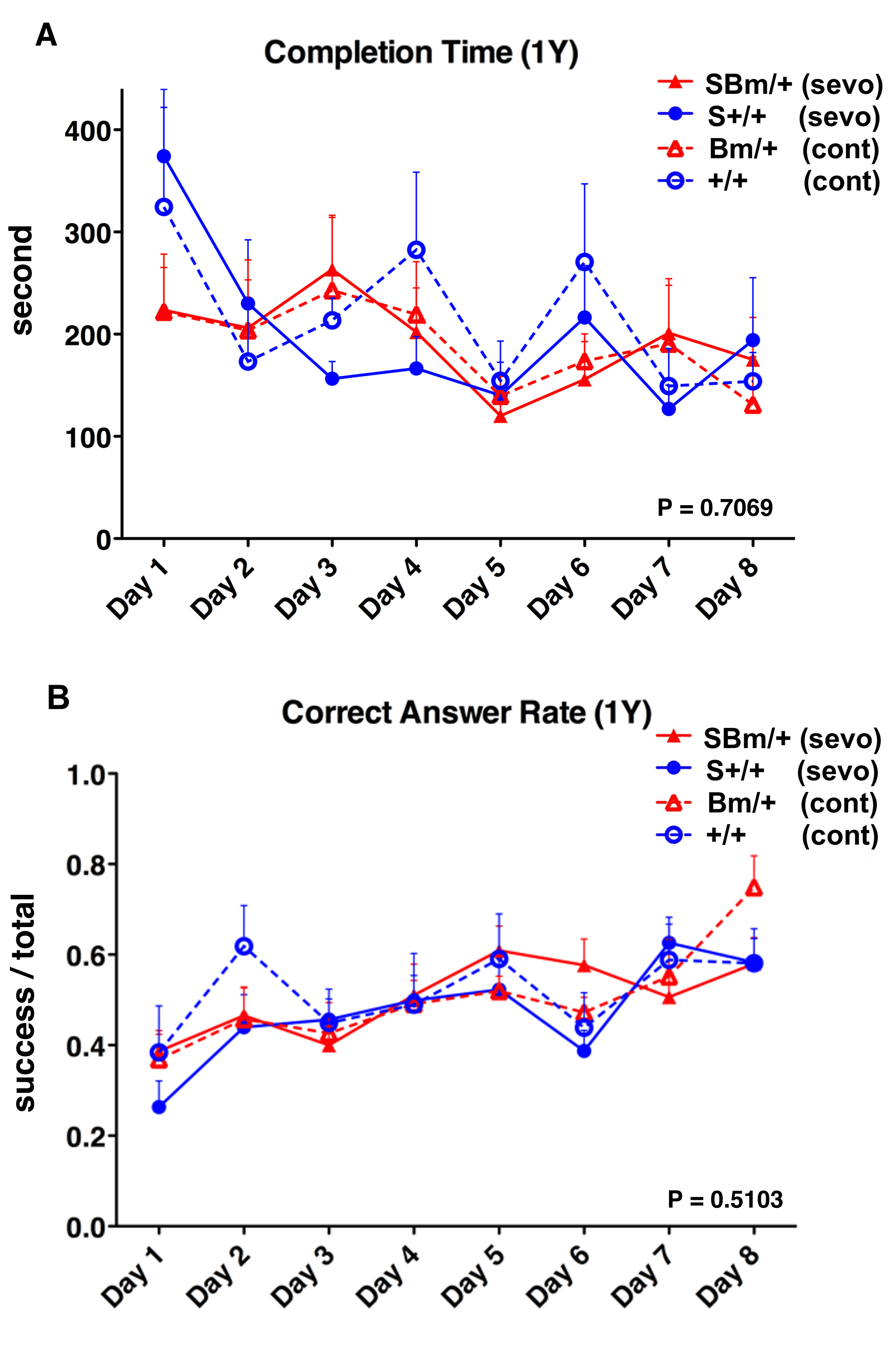

Supplement: FIGURE S3 — Eight-arm radial maze testing was performed in wild-type mice (+/+; blue lines) and mutant-BiP mice (Bm; red lines) at 1 year after fetal anesthetic exposure (S+/+, n = 9, closed circle; SBm, n = 14, closed triangle) and without exposure (+/+, n = 6, open circle; Bm, n = 8; open triangle). Tests were performed on 8 days. Significance was determined by one-way repeated measures ANOVA followed by Bonferroni’s multiple comparison testing. (A) Completion times are shown as the average value of each group + SEM. ANOVA, P = 0,7069 (B) Correct answer rates are shown as the average value of each group + SEM. The ratio of successful attempts to overall attempts was the correct answer rate (0.0–1.0). ANOVA, P = 0.5103. [file Image_3.tiff]
